# A Solar-Driven Oil–Water Separator with Fluorescence Sensing Performance

**DOI:** 10.3390/nano13192696

**Published:** 2023-10-03

**Authors:** Xin Li, Wei Lin, Florian Ion Tiberiu Petrescu, Jia Li, Likui Wang, Haiyan Zhu, Haijun Wang, Gang Shi

**Affiliations:** 1 Key Laboratory of Synthetic and Biotechnology Colloids, Ministry of Education, School of Chemical and Material Engineering, Jiangnan University, Wuxi 214122, China; lixin951001@163.com (X.L.); li951001@126.com (J.L.); lkwang@jiangnan.edu.cn (L.W.); bbszhy1979@163.com (H.Z.); 2 Department of Mechanisms and Robots Theory, National University of Science and Technology Polytechnic Bucharest, 060042 Bucharest, Romania; fitpetrescu@gmail.com

**Keywords:** evaporator, oil–water separation, fluorescence, photothermal conversion

## Abstract

Presently, the separation of oil and water through functional membranes inevitably entails either inefficient gravity-driven processes or energy-intensive vacuum pressure mechanisms. This study introduces an innovative photothermal evaporator that uses solar energy to drive oil–water separation while concurrently facilitating the detection of Fe^3+^ in wastewater. First, by alkali delignification, small holes were formed on the side wall of the large size tubular channel in the direction of wood growth. Subsequently, superhydrophilic SiO_2_ nanoparticles were in situ assembled onto the sidewalls of the tubular channels. Finally, carbon quantum dots were deposited by spin-coating on the surface of the evaporator, paralleling the growth direction of the wood. During the photothermal evaporation process, the tubular channels with small holes in the side wall parallel the bulk water, which not only ensures the effective water supply to the photothermal surface but also reduces the heat loss caused by water reflux on the photothermal surface. The superhydrophilic SiO_2_ nanoparticles confer both hydrophilic and oleophobic properties to the evaporator, preventing the accumulation of minute oil droplets within the device and achieving sustained and stable oil–water separation over extended periods. These carbon quantum dots exhibit capabilities for both photothermal conversion and fluorescence transmission. This photothermal evaporator achieves an evaporation rate as high as 2.3 kg m^−2^ h^−1^ in the oil–water separation process, and it has the ability to detect Fe^3+^ concentrations in wastewater as low as 10^−9^ M.

## 1. Introduction

As is well known, more and more industries produce a large amount of oily wastewater, making it difficult for people to obtain enough clean water [1,2]. Due to the small size of the emulsion droplet (<20 μm) and its easy deformation, the efficient separation of oil–water emulsions is considered a challenging task and one with great urgency [3]. Many separation techniques have been developed in industry, such as filtration [3], centrifugation [4] and precipitation [5], but these methods are inefficient and time-consuming.

In recent years, membrane separation technology has been widely used in the separation of oil–water emulsions due to its advantages of high separation efficiency and high separation selectivity. At present, there are two main membrane functional materials used for oil–water separation, including the superhydrophobic membrane [6,7] and the superhydrophilic membrane [8,9]. The superhydrophobic membrane usually possesses special micro- and nano-structure, which can form a high contact angle on its surface. When the oil–water emulsion to be separated passes through the superhydrophobic membrane, under certain pressure, the oil molecules will be adsorbed on the surface of the superhydrophobic membrane, and they will enter the other side along the direction of the micro- and nano-structure through the membrane hole. This is due to the interaction between its surface tension and the surface of the superhydrophobic membrane. In addition, water molecules can neither wet the surface of the superhydrophobic membrane nor enter the other side of the membrane through the pores, thus achieving efficient oil–water separation. Zhang et al. [10] reported a biomimetic nanofiber membrane with underwater superhydrophobicity and antifouling properties. Water in oil emulsion with droplet size ranging from nanometer to micrometer was successfully separated, which showed good flux (~12,994 L·m^−2^·h^−1^) and high separation efficiency (water content of the filtrate was less than 50 ppm). However, due to their inherent lipophilicity, superhydrophobic membrane can lead to the blockage of membrane pores during long-term use, which is one of the key limitations resulting in low permeability flux and high transmembrane pressure [11,12]. Compared with the superhydrophobic membrane, the superhydrophilic membrane has a strong hydrophilicity, which can quickly adsorb water molecules and form a hydration layer to provide a spatial exclusion barrier so as to better prevent oil droplets from entering the membrane pores and achieve efficient oil–water separation. For example, by using electrospinning/electrospraying technology, Sun et al. [13] obtained a superwetting membrane (PAN@PP FM) with a hierarchical structure that has a water contact angle of 0° and an underwater oil contact angle of 166°, allowing for the effective separation of oil and water under the action of gravity. In addition, due to the high porosity and the interconnection between the pores, the membrane can separate different types of oil and water with high throughput, excellent oil discharge efficiency and excellent anti-fouling performance. However, the above methods are driven by gravity or vacuum pressure to achieve the separation of oil–water emulsions, which has the limitations of high energy consumption and long separation time.

In previous reports, CDs and wood have been applied to solar interface water evaporation as photothermal materials and water-transferring materials, respectively [14,15]. However, no work has been reported on CDs and wood being applied to solar-driven oil–water separation. The operation of a solar-driven oil–water separator relies on clean and renewable solar energy, which can reduce the utilization of fossil fuels and the cost of water purification. Here, a solar-driven oil–water separator was designed in this study, which was obtained by removing lignin from the wood, modifying carbon quantum dots on the upper surface and constructing superhydrophilic structures on the lower surface. Compared with the traditional membrane separation technology, this oil–water separator, which can combine good thermal management, efficient photothermal conversion and good underwater oil drainage, can avoid the implementation of pressure in the oil–water separation process of emulsion, which has better economic benefits and applicability. In addition, the photothermal layer containing carbon quantum dots has fluorescence sensing ability, achieving the removal and fluorescence detection of Fe^3+^ in wastewater.

## 2. Materials and Methods

### 2.1. Materials

Tannin (TA), citric acid monohydrate (CA), ethanol (CH_3_CH_2_OH), anhydrous ethylenediamine, sodium dodecyl sulfate (SDS), y-amino-propyl triethoxy silane (APTES), polyvinyl alcohol 1799 (PVA), dichloromethane, sodium sulfite (Na_2_SO_3_), hydrogen peroxide (H_2_O_2_) and sodium hydroxide (NaOH) were purchased from Sinopharm Chemical Reagent Co., Ltd., Shanghai, China. All reagents were used directly without further purification. Balsa was obtained from Yichuang, Hebei, China.

### 2.2. Fabrication of Delignified Wood Blocks

Balsa wood blocks (26 mm × 20 mm × 5 mm) were immersed in a mixed aqueous solution containing 2.5 M NaOH and 0.4 M Na_2_SO_3_. The solution was brought to a boil at 100 °C for 7 h. Subsequently, the wood blocks were rinsed with deionized water. The treated wood blocks were then soaked in a boiling 2.5 M H_2_O_2_ solution until they turned white, which was designated as delignified wood (DLW) [16,17,18].

### 2.3. Fabrication of DLW Modified by SiO_2_ Nanospheres

Initially, a mixed solution of Tris-HCl (pH = 8.5) and ethanol in a volumetric ratio of 5:1 was prepared. The DLW was then submerged in this mixed solution and subjected to ultrasonication for 10 min. Next, 50 mg of TA and 60 mg of APTES were dissolved in 20 mL of Tris-HCl solution and 4 mL of ethanol. The pretreated DLW was subsequently immersed in the TA and APTES mixture at room temperature for 6 h, resulting in SiO_2_ nanosphere-modified DLW, which was denoted as TPDLW. The TPDLW was then sonicated in deionized water for 1 h to thoroughly remove surface impurities [19].

### 2.4. Fabrication of Carbon Quantum Dots (CDs) 

The typical synthesis of CDs is as follows: 1.0507 g of hydrated citric acid and 335 μL of anhydrous ethylenediamine were successively added in 10 mL of ultrapure water. The solution was subjected to ultrasonication for 30 min and then underwent a hydrothermal reaction at 200 °C for 5 h. Finally, the product was dialyzed (1000 D) for one week and subsequently freeze-dried to obtain carbon quantum dot powder [20,21].

### 2.5. Fabrication of v-TPDLW@CDs@PVA Evaporator

Initially, 1 g of PVA was dissolved in 18 g of deionized water at 90 °C for 3 h. Subsequently, 5 mg of CDs was dissolved in 1 mL of the obtained PVA solution. Finally, the v-TPDLW@CDs@PVA evaporator was generated when the solution was spin-coated onto the TPDLW surface in the direction of the tree growth at a speed of 5000 rpm. The h-TPDLW@CDs @PVA evaporator was generated using a similar method, except that the solution was spin-coated on the cross-section of TPDLW.

### 2.6. Preparation of Oil-in-Water Emulsion

Water and dichloromethane were mixed in a volume ratio of 9:1 with 0.1% weight as SDS. The mixture was continuously stirred for 24 h to yield a white emulsion.

### 2.7. Analysis of Water Evaporation Performance

The oil-in-water emulsion was put in a container with a 25 mm inner diameter. The emulsion was transferred to the bottom of the v-TPDLW@CDs@PVA evaporator by a cotton thread. Solar evaporation experiments were conducted under simulated sunlight, with a CEL-HXF300 xenon lamp (China Education Au-light, Beijing, China) providing a light intensity of 1 KW m^−2^. The change in water evaporation mass was recorded at 5 min intervals, and the water evaporation rate was calculated based on the water evaporation. The light irradiation area (26 mm × 20 mm) and water transfer thickness (5 mm) of all types of evaporators were the same. The entire experiment was conducted at an ambient temperature of 25 °C and a humidity of 60%.

### 2.8. Characterization

Transmission electron microscopy (TEM, JEM-2100 plus, JEOL, Tokyo, Japan), scanning electron microscopy (SEM, S-4800, Hitachi, Tokyo, Japan) and ultra-depth three-dimensional microscopy (VHX-1000C, Keyence, Osaka, Japan) were used to observe the morphology of the samples. The solar water evaporation was performed under the radiation of the 300 W xenon lamp (CEL-HXF300, CEAuLight, Beijing, China) as a visible light source. The surface temperature of the samples was measured by an infrared camera (FLIR E6). The photoluminescence (PL) spectra were measured at room temperature on a fluorescence spectrophotometer (FS5, Edinburgh Instrument Company, UK) with an excitation wavelength of 340 nm. Surface wettability was characterized by using a contact angle measurement system (OCA 40, Dataphysics Company, Filderstadt, Germany).

## 3. Results and Discussion

### 3.1. Fabrication Process and Morphological Characterization

**Figure 1:**
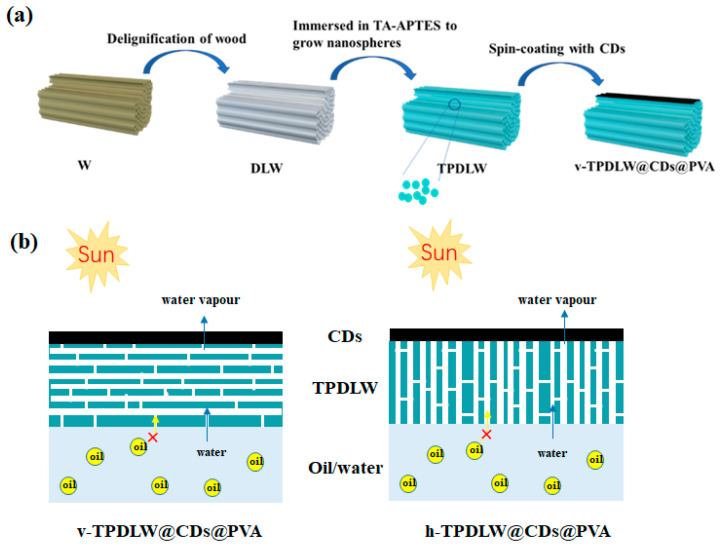
(**a**) Scheme of fabricating v-TPDLW@CDs@PVA evaporator; (**b**) scheme of solar oil–water separation of v-TPDLW@CDs@PVA and h-TPDLW@CDs@PVA.

**Figure 2:**
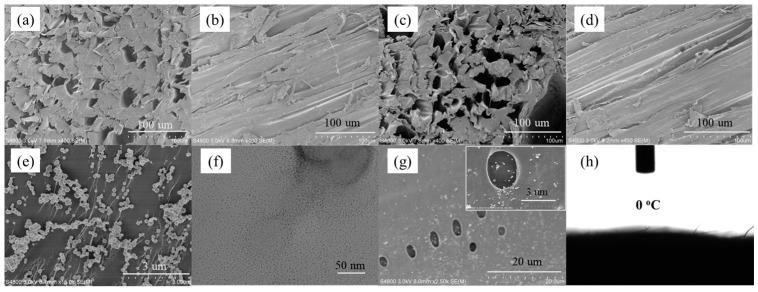
SEM images of different structures: (**a**,**b**) the channel structure of W; (**c**,**d**) the channel structure of DLW; (**e**) SiO_2_ nanoparticles; (**f**) TEM images of CDs; (**g**) SEM images of v-TPDLW@CDs@PVA, the illustration is an enlarged version of the corresponding SEM image; (**h**) the contact angles of v-TPDLW@CDs@PVA.

The fabrication process of the v-TPDLW@CDs@PVA evaporator is illustrated in Figure 1a. Initially, the wood (W) was subjected to an alkali treatment to eliminate lignin. Subsequently, hydrogen peroxide bleaching was employed to further remove residual lignin and hemicellulose, resulting in DLW. Microscopic morphology of the inner trunk, both perpendicular (Figure 2a) and parallel (Figure 2b) to its growth direction, reveals the presence of cylindrical channels with diameters ranging from 10 to 40 μm, exhibiting smooth and dense channel walls. Alkali treatment leads to an increase in the number of channels within the trunk, accompanied by the formation of 1–5 μm pores on the surface of channel wall, as depicted in Figure 2c,d. These channels can not only provide water transport channels but can also improve light absorption by increasing the number of light reflections [22,23]. Subsequently, DLW was immersed in a mixed solution of tannic acid and aminopropyltriethoxysilane (TA/APTES) for silane functionalization. The resulting SiO_2_ nanoparticles were bonded onto DLW channels, generating TPDLW. The SiO_2_ nanoparticles, uniformly distributed on the channel walls’ surface, possess sizes ranging from 100 to 200 nanometers, as shown in Figure 2e. These SiO_2_ nanoparticles establish a robust connection with TA adsorbed on the microchannel walls, forming a secure binding. Finally, a solution of PVA with embedded CDs was spin-coated onto the surface of TPDLW, aligned parallel to the tree growth direction, thereby obtaining the v-TPDLW@CDs@PVA evaporator. The CDs were uniformly dispersed on the microchannel walls of TPDLW, with particle diameters measuring only 3–4 nm, as depicted in Figure 2f,g. The solar-driven oil–water separator of v-TPDLW@CDs@PVA and h-TPDLW@CDs@PVA are shown in Figure 1b. The tubular channels of v-TPDLW@CDs@PVA are parallel to the bulk water surface, and the tubular channels of h-TPDLW@CDs@PVA are perpendicular to the bulk water surface. The tubular channels within the evaporator run parallel to the bulk water, while the hole on the channel walls are oriented perpendicular to the bulk water. This arrangement ensures ample water transport to the evaporator surface for efficient evaporation, simultaneously extending the water’s transport pathway to reduce thermal losses caused by surface-heated water reflux back into the bulk water.

### 3.2. Verification of the Advantages of Evaporator Design

**Figure 3:**
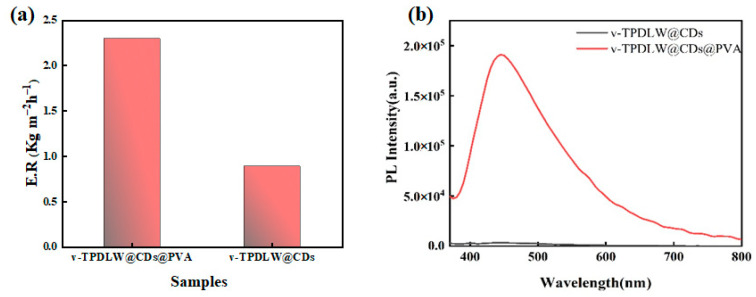
(**a**) Evaporation rate and (**b**) fluorescence spectra of v−TPDLW@CDs and v−TPDLW@CDs@PVA.

In order to demonstrate the design advantages of the proposed evaporator, v-TPDLW@CDs@PVA was compared with v-TPDLW@CDs and h-TPDLW@CDs@PVA. CDs are not only good photothermal materials, but they are also fluorescent materials that are responsive to metal ions [24,25,26,27,28]. The results of the comparisons indicate that the water evaporation rate of v-TPDLW@CDs@PVA is much higher than those of v-TPDLW@CDs, as shown in Figure 3a. The PVA has a good bonding effect, which can increase the load of CDs on the evaporator surface. On one hand, it can enhance the efficiency of sunlight absorption, and then improve the photothermal capacity of the evaporator. On the other hand, the hydrogen bonding between PVA and water can reduce the enthalpy of water evaporation [29], which can increase the water evaporation rate of the evaporator. Meanwhile, the fluorescence intensity of v-TPDLW@CDs@PVA is much higher than that of v-TPDLW@CDs, as shown in Figure 3b. PVA can effectively prevent the accumulation of CDs. However, high load of CDS can improve the absorption of excitation light with single-wavelength fluorescence, and thus improve its fluorescence intensity. In the absence of PVA, CDs resided on the wood surface, with surface oxygen functional groups prone to aggregation, inducing non-radiative energy transfer and leading to fluorescence quenching in v-TPDLW@CDs. Conversely, for v-TPDLW@CDs@PVA, the presence of hydroxyl groups in PVA facilitated hydrogen bonding with CDs, which increases the distance between CDs and thus reduces the probability of fluorescence quenching [30,31].

**Figure 4:**
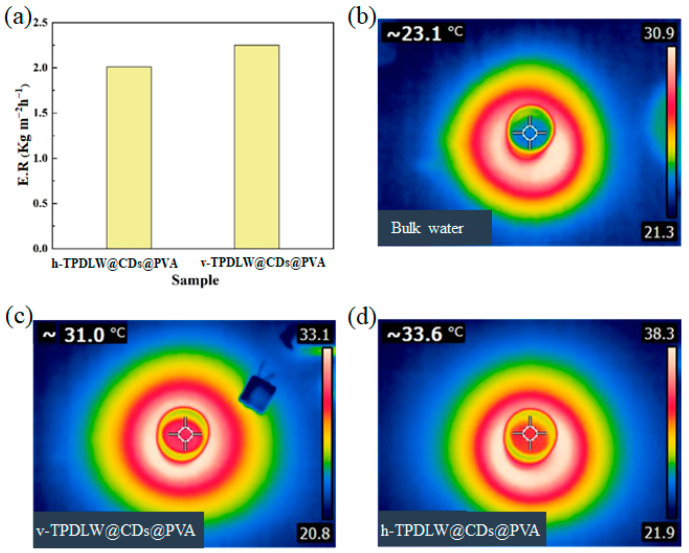
(**a**) Evaporation rate of h−TPDLW@CDs@PVA and v−TPDLW@CDs@PVA; (**b**) surface water temperature of bulk water; (**c**) surface water temperature of v−TPDLW@CDs@PVA; (**d**) surface water temperature of h−TPDLW@CDs@PVA after 1 h of illumination.

Figure 4 shows that the evaporation rate of v-TPDLW@CDs@PVA surpasses that of h-TPDLW@CDs@PVA. Before the simulation of solar radiation, the temperature of the water on the evaporator surface was 23.1 °C. After simulating solar illumination for 1 h, the surface temperatures of v-TPDLW@CDs@PVA and h-TPDLW@CDs@PVA reached 31 °C and 33.6 °C, respectively. In comparison to h-TPDLW@CDs@PVA, v-TPDLW@CDs@PVA exhibits a certain thermal insulation advantage. Specifically, within v-TPDLW@CDs@PVA, water was rapidly diffused laterally through many tubular channels, while the evaporating surface of the evaporator was wetted longitude-wise by capillary water absorption through holes in the side walls of the channels during the process of evaporation. When the hot water on the surface of the evaporator returns downward, this structure extends the return path and reduces the heat loss of the water backflow. Although the tubular channels in h-TPDLW@CDs@PVA are oriented perpendicular to the bulk water, offering a rapid upward path for water transport and steam escape, this swift water transfer also leads to increased thermal losses due to backflow [32,33].

### 3.3. Oil–Water Separation Performance

**Figure 5:**
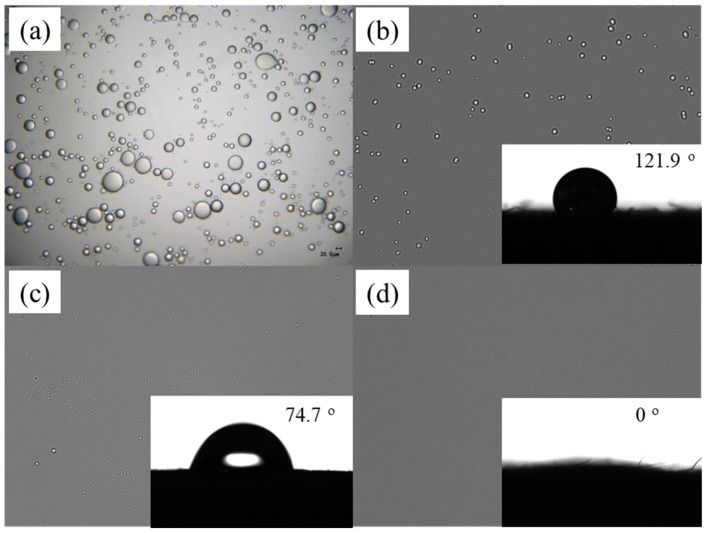
Oil–water separation effect of different samples: (**a**) original oil–water emulsion; (**b**) v-W@CDs@PVA; (**c**) v-DLW@CDs@PVA; (**d**) v-TPDLW@CDs@PVA. The internal illustrations show the water contact angle of the corresponding wood structure in the air.

Figure 5 has shown the oil–water separation capability of v-TPDLW@CDs@PVA in solar-driven water vapor processes, comparing it with v-W@CDs@PVA and v-DLW@CDs@PVA. It has been observed by optical microscopy that the size of droplets in the newly prepared emulsified oil–water mixture ranges from tens to hundreds of micrometers (Figure 5a). The condensate collected after oil–water separation through different solar water evaporators is shown in Figure 5b,c. Post-evaporation treatment with v-W@CDs@PVA and v-DLW@CDs@PVA still exhibits the presence of residual small-sized oil droplets in the collected water phase. In contrast, no oil droplets are discerned in the collected water phase following the evaporation treatment with v-TPDLW@CDs@PVA. This indicates that the v-TPDLW@CDs@PVA evaporator can effectively prevent oil droplets from entering the evaporator, thereby achieving effective oil–water separation. However, due to the fact that dichloromethane can be partially dissolved in water, the condensed water collected after evaporation may contain low concentrations of dichloromethane. To elucidate this phenomenon, water contact angle measurements were conducted on the surface contacting with the lotion, as shown in the insets of Figure 5b,c. The contact angle at the lower surface of v-W@CDs@PVA was 121.9°, and it takes 67 s for the water droplets to spread completely. Conversely, the contact angle at the lower surface of v-DLW@CDs@PVA was 74.7°, and the water droplets can be completely spread within 8 s. Remarkably, the contact angle at the lower surface of v-TPDLW@CDs@PVA was reduced to as low as 0°, and the water droplets spread out rapidly within 1 s, thereby showing exceptional superhydrophilic capabilities. This phenomenon is attributed to the potent water-capturing capacity of the hydrophilic nanospheres on the lower surface of v-TPDLW@CDs@PVA. This remarkable superhydrophilic property facilitates the formation of a stable hydration layer on the sample surface, significantly enhancing its oil-repelling performance in water, consequently promoting oil–water separation [34].

**Figure 6:**
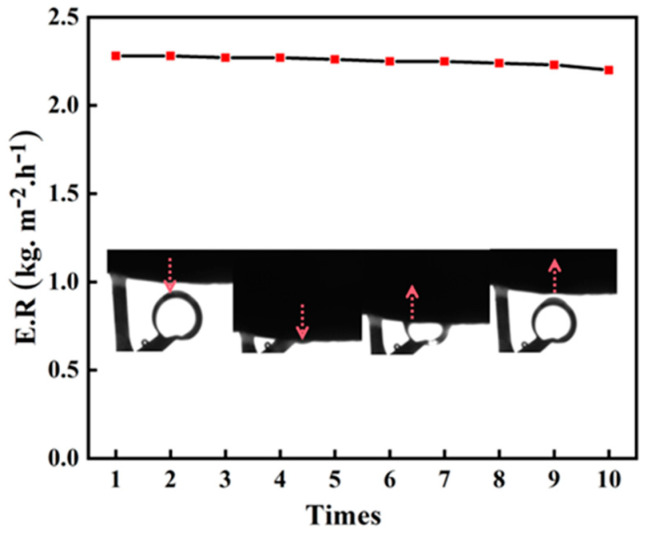
v-TPDLW@CDs@PVA cycling stability test and underwater oil repellency in oil–water emulsion.

In order to assess the durability of v-TPDLW@CDs@PVA, ten cycles of solar-driven oil–water separation experiments were conducted. The variations in water evaporation rates during this process were investigated, and the experimental results are shown in Figure 6. Following the completion of each testing cycle, the samples were cleaned with distilled water to prepare for subsequent oil–water separation experiments. Through ten cycles of testing, the water evaporation rate of v-TPDLW@CDs@PVA only exhibited a marginal decrease of 0.01 kg m^−2^ h^−1^, indicating remarkable durability. This suggests that v-TPDLW@CDs@PVA consistently maintains its effectiveness in efficiently separating water from oil–water emulsions over extended durations. Furthermore, after ten consecutive cycles of experimentation, an underwater oil contact angle assessment was performed on v-TPDLW@CDs@PVA. The specific experimental procedure involved placing an oil droplet in water, then gradually lowering the sample until complete contact was achieved between the sample and the oil droplet. Subsequently, the sample was slowly raised to achieve complete separation from the oil droplet. The changes in the oil contact angle are illustrated in the inset of Figure 6. After ten cycles of experimentation, v-TPDLW@CDs@PVA continued to exhibit robust underwater oleophobicity, validating its reliability over prolonged periods of oil–water separation processes.

### 3.4. Fluorescence Sensing of Fe^3+^

**Figure 7:**
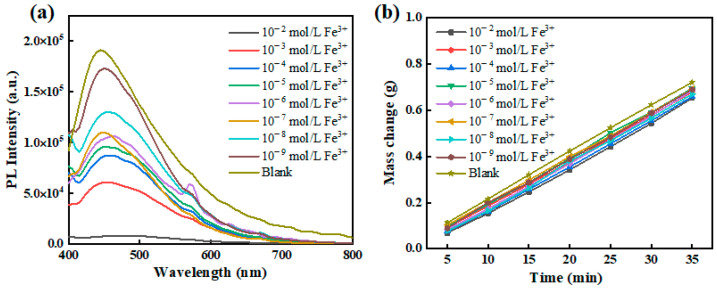
v-TPDLW@CDs@PVA at different concentrations of Fe^3+^: (**a**) fluorescence spectrum, (**b**) water evaporation rate.

Within v-TPDLW@CDs@PVA, the CDs serve a dual role as not only photothermal converters but also as fluorescent quantum dots endowed with a profusion of oxygen-bearing functional groups on their surfaces. Through coordination with Fe^3+^, CDs were fluorescence quenching, thereby enabling fluorescence-based sensing of Fe^3+^. In this context, v-TPDLW@CDs@PVA was employed for the photothermal evaporation of wastewater containing varying concentrations of Fe^3+^. During the evaporation process, driven by capillary forces, Fe^3+^ ions are continuously transported into the channel of v-TPDLW@CDs@PVA. Subsequently, these ions interact with the CDs on the channel surface through cooperative ligand bonding, leading to the attenuation of fluorescence intensity and enabling the recognition of Fe^3+^, as shown in Figure 7a. After 35 min of photothermal water evaporation, v-TPDLW@CDs@PVA demonstrates the capability to detect ultra-low concentrations of Fe^3+^ ions, reaching as low as 10^−9^ mol/L. This achievement in trace Fe^3+^ fluorescence detection is attributed to the capacity of photothermal water evaporation to concentrate Fe^3+^ ions onto the surface of the v-TPDLW@CDs@PVA evaporator. In addition, water evaporation rates of v-TPDLW@CDs@PVA for Fe^3+^ solutions of different concentrations were shown in Figure 7b. Before the action of Fe^3+^, the water evaporation rate of v-TPDLW@CDs@PVA stands at 2.33 kg m^−2^ h^−1^. Remarkably, upon exposure to varying concentrations of Fe^3+^, the water evaporation rate experiences only marginal reduction, ranging from 0.02 to 0.09 kg m^−2^ h^−1^. This phenomenon is attributed to potential coordination effects between Fe^3+^ ions and carbon quantum dots, which may influence the hydrogen bonding interactions between carbon quantum dots and water molecules. 

## 4. Conclusions

In conclusion, the v-TPDLW was formed by modifying superhydrophilic SiO_2_ nanospheres inside ligninized wood with a tannin-amino-siloxane reagent. The v-TPDLW@CDs@PVA evaporator which can separate oil and water was fabricated by spinning a CDs-PVA solution. The performance of water evaporation, oil–water separation and fluorescence sensing of v-TPDLW@CDs@PVA were studied. Notably, v-TPDLW@CDs@PVA demonstrates remarkable oil–water separation ability, maintaining excellent underwater oil-repellent capability over 10 cycles of water-assisted oil–water separation experiments, with a water evaporation rate as high as 2.3 kg m^−2^ h^−1^. Due to the efficient water evaporation of v-TPDLW@CDs@PVA, Fe^3+^ ions are enriched on the evaporative surface, enhancing the sensitivity of the substrate towards Fe^3+^ detection with a detection limit as low as 10^−9^ M. This research provides a new approach for oil–water separation. However, the v-TPDLW@CDs@PVA oil–water separator cannot be used again for ion detection after absorbing iron ions. How to solve this problem is worthy of further research.

## Data Availability

Not applicable.
